# A Novel Mutation in the ATP7B Gene: A Rare Manifestation of Wilson Disease With Liver Failure

**DOI:** 10.14309/crj.0000000000000977

**Published:** 2023-02-09

**Authors:** Rehmat Ullah Awan, Shazia Rashid, Ambreen Nabeel, Manesh Kumar Gangwani, Hrishikesh Samant

**Affiliations:** 1 Department of Medicine, Ochsner Rush Health System, Meridian, MS; 2 Department of Gastroenterology and Hepatology, Louisiana State University, Shreveport, LA; 3 Department of Medicine, University of Toledo Medical Center, Toledo, OH; 4 Department of Gastroenterology and Hepatology, Ochsner Health System, New Orleans, LA

**Keywords:** Wilson disease, acute liver failure, liver transplant, ATP7B gene mutation

## Abstract

Wilson disease is a hereditary disorder which involves anomalous copper metabolism. Typically, the presentation is systemic, involving vital organs such as the liver, kidney, and brain, among others. We report a unique case presenting with solitary organ involvement as acute liver failure with novel ATP7B gene mutation, which has never been reported before.

## INTRODUCTION

Wilson disease (WD) is a rare genetic condition characterized by excess copper buildup, which can affect virtually any organ system in the body. Owing to the presence of several genotypic and phenotypic variances, diagnosis and thus timely treatment can be challenging. Timely recognition and treatment can ensure a relatively normal life in patients with WD.

## CASE REPORT

A 16-year-old adolescent boy with no medical history presented to the hospital with right upper quadrant pain, nausea, and vomiting for 3 days. Family history was significant for a sister who died of hepatitis. He denied using alcohol, drug abuse, or Tylenol; of note, his parents immigrated from Honduras. On examination, he was hemodynamically stable and visibly jaundiced with marked scleral icterus; furthermore, he was also noted to be lethargic and somnolent. His laboratory test results were concerning for acute liver failure and acute kidney injury (Table 1). Further workup included genetic testing, alpha-1 antitrypsin, copper, ceruloplasmin, and iron studies. Serum toxicology was negative. Infectious and autoimmune workup was performed including anti-nuclear antibody and anti-smooth muscle antibody; Epstein-Barr virus, herpes simplex virus, cytomegalovirus, hepatitis panel, and varicella-zoster virus serologies; and total immunoglobulin (Ig) G and subclasses, IgA, IgM, soluble interleukin 2, and *Treponema cruzi*, which all came back negative other than low ceruloplasmin 6.0 mg/dL and high 24-hour urine copper excretion. Since admission, he was started on maintenance fluids and lactulose for encephalopathy. The patient was diagnosed with decompensated liver failure secondary to WD and thus was listed for liver transplant at a Model for End-Stage Liver Disease score of 39. He later underwent orthotropic liver transplant and did well postoperatively. Histopathological analysis of the patient's liver was also consistent with WD. Of note, his detailed genetic studies with whole-exome sequencing (WES) identified 2 likely pathogenic ATP7B variants (c.3446G>C and c.2355G>A), which were responsible for his disease (Table 2). The patient's laboratory test results normalized in subsequent clinic visits, and he improved considerably. Moreover, the patient and his entire family were also seen by a genetic counselor once he was discharged home.

**Table 1.** Admission laboratory test results

Laboratory values (reference range and units)	Patient result
White blood cell (4.50–13.50 k/μL)	18.69
Hemoglobin (13.0–16.0 g/dL)	5.7
Platelets (150–450 k/μL)	103
International normalized ratio (0.8–1.2)	4.0
Sodium (136–145 mmol/L)	130
Creatinine (0.5–1.4 mg/dL)	2.1
Blood urea nitrogen (5–18 mg/dL)	30
Albumin (3.2–4.7 g/dL)	2.1
Total bilirubin (0.1–1.0 mg/dL)	33.6
Direct bilirubin (0.1–0.3 mg/dL)	>14.0
Alkaline phosphatase (89–365 U/L)	21
Aspartate aminotransferase (10–40 U/L)	83
Alanine aminottransferase (10–44 U/L)	36
Ceruloplasmin (15.0–45.0 mg/dL)	6.0
Serum copper (665–1,480 μg/L)	1,195
Copper, urine, 24-hour μg/24 hr	4,237

**Table 2.** Causative variant(s) in disease genes associated with the reported phenotype

Gene	Disease	Mode of inheritance	Variant	Zygosity	Inherited form	Classification
*ATP7B*	ATP7B-related Wilson disease	Autosomal recessive	c.3446 G>C p.(G1149A)	Heterozygous	Father	Likely pathogenic variant
*ATP7B*	ATP7B-related Wilson disease	Autosomal recessive	c.2355 G>A p.(K785=)	Heterozygous	Unknown	Likely pathogenic variant

## DISCUSSION

WD is an autosomal recessive disease involving the ATP7B gene, which encodes for ATP7B protein involved in copper metabolism and excretion of excess copper into bile and plasma. In the absence of normal copper metabolism, toxic levels of copper builds up in tissues, which leads to direct cellular injury in the form of oxidative stress.^1^ If left untreated, affected patients can present with liver dysfunction, endocrinopathies, and neuropsychiatric conditions.^2^ Our patient's acute presentation is quite unique in comparison with the indolent course reported in the literature.

**Figure 1.**
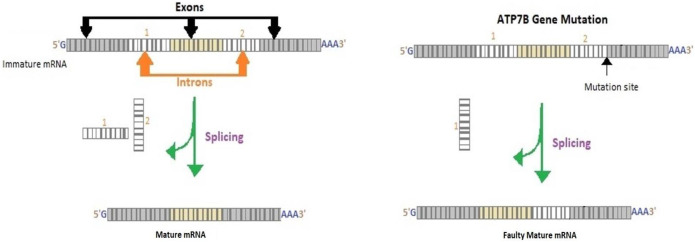
A diagrammatic representation of the mutated splicing site. Splicing is a process whereby introns (noncoding part) of mRNA are removed, allowing for joining of exons (coding part of mRNA) resulting in mature mRNA, which is then used for protein synthesis. The p.(K785=) mutation in the ATP7B gene destroys the splicing donor site, affecting downstream protein production leading to a dysfunctional copper metabolism. mRNA, messenger RNA.

There have been numerous studies focusing on mutations involving the ATP7B gene; around 1,019 mutations have been reported thus far.^3^ In our patient, the p.(G1149A) variant was inherited from the patient's father and has been reported before^4^; however, the p.(K785=) variant has not been previously published as pathogenic or benign to the best of our knowledge. This mutation alters the last nucleotide of the exon and is predicted to destroy the splice donor site and results in aberrant splicing (Figure 1). Moreover, it shows that this novel mutation is responsible for a highly dysfunctional protein resulting in accelerated hepatic failure, as seen in our patient. The fact that our patient exclusively had liver failure without any other organ system involvement makes this novel mutation crucial for gastroenterologists and hepatologists because this gene mutation caused targeted injury toward hepatocytes.

Treatment of WD hinges primarily on decreasing systemic copper load by chelation therapy, urinary excretion, and dietary restriction.^5,6^ In patients with cirrhosis, liver transplantation serves as a viable option with promising results as reported in the literature.^7^ Early treatment with copper chelators and zinc salts can not only halt the disease progression but may also prevent end-organ damage.^5^

## DISCLOSURES

Author contributions: RU Awan wrote the mansucript and is the corresponding author. S. Rashid edited and revised the manuscript. A. Nabeel review of literature and referencing. MK Gangwani review of literature and editing. H. Samant reviewed and edited final version and is the senior editor.

Financial disclosure: None to report.

Informed consent was obtained for this case report.
